# Approaches to Interrogating the Human Memory B-Cell and Memory-Derived Antibody Repertoire Following Dengue Virus Infection

**DOI:** 10.3389/fimmu.2019.01276

**Published:** 2019-06-06

**Authors:** Zoe L. Lyski, William B. Messer

**Affiliations:** Department of Molecular Microbiology and Immunology, Oregon Health and Sciences University, Portland, OR, United States

**Keywords:** dengue, virus, hybridoma, B-cell immortalization, ELISPOT, flow cytometry, long-term immunity, monoclonal antibody

## Abstract

Memory B-cells (MBCs) are potential antibody secreting immune cells that differentiate and mature following host exposure to a pathogen. Following differentiation, MBCs remain in peripheral circulation after recovery and are poised to secrete antigen-specific antibodies if and when they are re-exposed to their cognate antigen. Consequently, MBCs form the founder population and provide one of the first lines of pathogen-specific defense against reinfection. The role MBCs play is complicated for viruses that are heterologous, such as dengue virus (DENV), which exist as antigenically different serotypes. On second infection with a different serotype, MBCs from initial dengue infection rapidly proliferate and secrete antibodies: many of these MBC derived antibodies will be cross-reactive and weakly neutralizing, while some antibodies may recognize epitopes conserved across serotypes and have the capacity to broadly neutralize 2 or more serotypes. It is also possible that a new population of MBCs and antibodies specific for the second virus serotype need to arise for long-term broader immunity to develop. Methods to interrogate and track memory B cell responses are important for evaluating both natural immunity and vaccine response. However, the low abundance of MBCs for any specific pathogen makes it challenging to interrogate frequency, specificity, and breadth for the pathogen of interest. This review discusses current approaches that have been used to interrogate the memory B cell immune response against viral pathogens in general and DENV specifically. Including strengths, limitations, and future directions. Single-cell approaches could help uncover the DENV specific MBC antibody repertoire, and improved methods for isolating DENV specific monoclonal antibodies from human peripheral blood cells would allow for a functional analysis of the anti-DENV repertoire.

## Introduction

Neutralizing antibody responses play a critical role in anti-viral immunity–controlling and preventing infection, and are an important aim of vaccination. During initial infection, naïve host B-cells, specific to the infecting antigen, proliferate and differentiate into short-lived plasmablasts that secrete antibodies at a high rate to combat the existing infection. Following viral clearance, two distinct layers of humoral immunity remain to protect against repeat infection with the same antigen—antibodies in the sera, constitutively produced by long lived plasma cells (LLPCs) and memory B-cells (MBC) primed to expand and secrete antibodies upon antigen re-exposure.

LLPCs, are terminally differentiated, non-dividing cells that reside in the bone marrow and produce antibodies for years to decades (1–3) providing protection against repeat infections with the same antigen (4, 5). These antibodies are typically assessed by *in vitro* neutralization and binding assays, and in many cases regarded as correlates of protection against viral pathogens (6).

MBCs make up the second line of antibody-mediated defense, providing protection by rapidly activating, proliferating and secreting antibodies in response to cognate antigen. Once regarded as a backup to LLPC-derived antibodies, the specificity and breadth of potential MBC responses are increasingly appreciated, especially with regard to protection against heterogeneous but antigenically related viral pathogens, such as influenza, different serotypes of DENV, and viral escape mutants (5, 7).

Naïve B-cells, MBC precursors, originate in the bone marrow before migrating to the spleen where they undergo further differentiation, redistribute to lymph nodes, and await antigen encounter. Recent work in mice suggests that MBCs originate in low affinity germinal center compartments within peripheral lymph nodes (5), which might contribute to more broadly reactive Ig receptors and increase the breadth of recall responses (5, 7). MBCs form a heterogeneous population, and it is thought that they preferentially differentiate upon reinfection. Specifically, IgG MBCs favor differentiating into plasmablasts whereas IgM MBCs preferentially re-enter germinal centers to undergo further rounds of affinity maturation (5, 8–10).

MBCs can be identified by their B-cell receptor (BCR), a membrane bound immunoglobulin (Ig) identical to the antibody they secrete upon activation. Upon reinfection, the recall response is rapid, dominated by high affinity isotype switched antibodies, IgG, IgA, or IgE, depending on pathogen. This recall response leads to the generation of new antigen-specific LLPCs and MBCs (5).

Although human MBCs have been characterized for many important viral pathogens, including HIV (11), RSV (12), influenza (13), human DENV MBC derived antibodies were not fully characterized until 2010 (14, 15). Mosquito-transmitted DENV is responsible for ~100 million symptomatic cases and 35,000 deaths annually (16) making it the most common and serious vector-borne disease affecting humans. DENV is an enveloped positive sense RNA virus, that circulates as four distinct serotypes (DENV 1-4), with 60–85% shared sequence homology (17). Repeat infections often occur in DENV endemic regions–Asia, Latin America, Africa, and parts of Oceana. Population growth, increased global travel, and spread of the vector have led to increasing epidemics. DENV infection causes symptoms that include high fever and rash. A portion of patients (~500,000 per year) develop severe dengue—referred to as dengue shock syndrome (DSS) or dengue hemorrhagic fever (DHF)—which can further progress to organ failure and death. First (1°) infection with one serotype is thought to provide life-long protection against that serotype, but only short-lived protection against heterologous infection (18). Secondary (2°) infection with a different serotype can lead to broader protection, up to all four serotypes, but comes at a greater risk of serious disease during acute infection, through a process of antibody dependent enhancement (19), which occurs when sub-neutralizing antibodies bind to virus and facilitate uptake into cells via Fc receptors (20). The mechanism by which subsequent broader immunity develops is incompletely understood: while it is known that immediately following second infection the antibody response contains a large proportion of cross-reactive antibodies that can neutralize both viruses (21) the relative contribution of type specific, weakly cross-neutralizing and broadly neutralizing antibodies to long-term immunity is unclear, and may depend on virus maturation state (22). Further complicating the hypothesized role of broadly cross-neutralizing antibodies is the recent finding by Raut et al. (23) that *in vitro* neutralization assays using mature and partially mature tissue-culture derived DENV1 over-estimated by almost 15-fold the potency of heterotypic neutralizing antibodies when compared to neutralizing potency against the same fully mature DENV1 circulating in humans. Deeper understanding of the diversity and epitope-specificity of 1° MBCs could lead to the development of subunit vaccines that preferentially elicit potently neutralizing antibodies against all 4 DENV serotypes while avoiding potentially enhancing antibodies.

Analyses of human antibody response to DENV infection have traditionally characterized serum antibodies, a product of LLPCs through virus neutralization, binding, and enhancement assays (24). More recently, greater focus has been put on characterizing B-cells that produce these antibodies. Historically, methods to interrogate antigen-specific B-cells, particularly in humans, have been challenging to develop (25, 26). However, complex, studying individual and population MBCs and the monoclonal antibodies (mAbs) they produce is an area of critical importance. Such studies allow for better understanding of the nature of human immune response to pathogens such as DENV, and are expected to lead to more rationally designed vaccines.

Over several decades, methods to interrogate antigen-specific MBCs have had several useful functions: identifying subset of MBCs available to respond to repeat infections (27, 28), tracking MBCs prior to and after vaccination or booster (6), isolating and characterizing human mAbs following natural infection or vaccination (29), and analyzing memory-derived antibody repertoires (28). Only recently have these methods been employed in the DENV field. Here we review the leading approaches for characterizing human DENV MBCs, evaluating their strengths, limitations and potential for further contribution to the field (summarized in Table 1).

**Table 1:** Summary of human DENV-specific monoclonal antibodies isolated from immune donors.

**References**	**Method**	**Efficiency**	**#Donors**	**mAbs isolated**	**Key findings**
Schieffelin et al. (15)	B-cell immortalization (EBV)	N/A	1 > 2 years post infection	3	DENV-specific MBCs were in circulation >2 years post exposure 2% of cultures were DENV2 specific All isolated mAbs were IgG1
Beltramello et al. (14)	B-cell immortalization (EBV) with CpG Screened and cloned by limiting dilution.	6.5–14%	5 3-1° 2-2° 200 days to >8 years post-infection	70	All of the isolated mAbs were IgG: 68 IgG1, 1 IgG3, 1 IgG4 13 mAbs recognized EDIII and were the most potently neutralizing of all the mAbs isolated (5 serotype specific, 8 cross reactive) 34 mAbs recognized DI/DII they were highly cross reactive and less neutralizing, 6 mAbs recognized PrM, 11 mAbs recognized non-structural proteins, and 1 recognized capsid
Smith et al. (27)	B cells immortalized (EBV) with CpG and CHK2. Screened by ELISA. Positive wells fused to generate hybridomas.	>10-fold increase in the # of successful human hybridomas generated	12 6–1° 6–2° 4–24 years post infection	37	29/37 isolated mAbs recognized E protein, 26 were IgG1 and 3 were IgG2 26/37 isolated mAbs were cross reactive, most bound to EDI/II 5- isolated mAbs EDIII specific (1C7, 1M23, 2J20, 1B23, 1M19) all cross reactive 3-mAbs had moderate to strong neutralizing potency against at least one serotype (2D22, 5J7, 2J20) 8- isolated mAbs PrM specific, mAbs exhibited enhancing properties
Smith et al. (30)	B-cell immortalization (EBV) CpG and CHK2 Screened by ELISA and neutralization then fused to generate hybridomas	N/A	3 2–1° 1–2° 1–9 years post-infection	50	Most potently neutralizing mAbs bound to EDIII (1M7) or complex (1F4) epitopes on intact virions. DENV specific MBC frequency similar between primary and secondary donors at 14–18 DENV specific MBC per thousand B cells. 15 of the isolated mAbs were non-neutralizing, and bound to rE or PrM.
Cox et al. (25)	FACS using E (DENV2-80E) and dual labeled secondary antibodies Isolated double positive MBCs	20 million PBMCs, 148 DENV E+ MBCs sorted.	1 from an endemic region Serum neutralized all 4 serotypes	9	DENV E specific MBCs are present in naturally infected donors. Authors isolated and characterized DENV neutralizing mAbs from MBCs against envelope domain I and the fusion loop. Of the sorted MBCs following 2 week stimulation in culture 64% were positive for IgG, 20% were positive for DENV by ELISA, 8% secreted DENV2 specific mAbs Of the 9 mAbs isolated 1-non-neutralizing, 3-serotype-specific, 2-neutralized 2–3 serotypes 3-neutralized 4 serotypes.
Appanna et al. (28)	FACS using fluorescently labeled DENV3. Isolated DENV positive MBCs	N/A	4 2°	19	40–60% of the DENV-specific MBCs sorted bound to DENV Most mAbs isolated bound to complex epitopes, 24.4% bound to PrM and 17.8% bound to rE Majority were cross reactive and weakly neutralizing
Nivarthi et al. (31)	B-cell immortalization (EBV) CpG and CHK2 followed by fusion	N/A	2 1° DENV-4	8	Frequency of DENV-specific B-cells in circulation 0.19–0.2%. Of the 8 mAbs isolated, 2 neutralized DENV-4 and recognize regions on EDI/EDII hinge.

### Limiting Dilution Assay (LDA)

The LDA was first used to detect virus-specific MBCs in mice over 20 years ago (32). This approach allows the frequency and specificity of rare antigen-specific MBCs in circulation to be enumerated. PBMCs or enriched B-cells are stimulated *ex vivo* with a mitogen cocktail along with non-proliferating feeder cells. With this approach MBCs become antigen-secreting cells. The cells can be enumerated by ELISpot (described later) or secreted antibodies assayed by antigen-specific ELISA. This approach has been used to determine the frequency of viral-specific MBCs in humans following vaccination or natural infection (6, 33–35).

### Strengths and Limitations

Non-specific stimulation of human MBCs allows for the characterization of multiple antigen-specific MBC derived antibodies from a single PBMC sample (6). Antibody containing supernatant or MBCs can be used for a wide range of assays including: ELISpot, ELISA, and neutralization. The major limitations of this approach are that the cells are not immortalized therefore longevity is limited, surface BCR is down-regulated, and single antigen-specific MBCs clones cannot be identified and subjected to downstream sequencing and cloning.

### Enzyme-Linked Immunosorbent Spot Assay (ELISpot)

Provides a sensitive and specific tool to detect antigen-specific MBCs. First described over 35 years ago (36) as a method for quantifying rare B and T cells and is still widely used today, as it is sensitive enough to detect a single antigen-specific cell. Plasmablasts can be studied directly *ex vivo*, but MBCs must be stimulated to become antibody-secreting cells. Membrane-bound antigen enables binding of mAbs secreted by B-cells. Bound antibody is detected using a secondary antibody and a colorimetric substrate, resulting in colored spots on the membrane that can be easily enumerated using imaging software. Advances in ELISpot technology have allowed researchers to detect different isotypes of MBCs that recognize multiple epitopes and multiple antigens (37). Recently developed multifunctional FluoroSpot assays allow enumeration of cross-reactive and type-specific DENV and Zika MBCs following natural infection and vaccination (19, 38). This allows researchers to determine serotype specificity on a single-cell basis, rather than polyclonal level.

### Strengths and Limitations

ELISpot is highly sensitive and allows for the enumeration of rare cells of interest—frequency, specificity, and antibody isotype can be determined. The major limitation is that it does not allow for isolation and downstream analysis—Functional properties of antibodies, such as neutralization cannot be assessed, and cells' BCRs of interest cannot be sequenced or cloned for mAb production.

### Hybridoma Approaches

The use of hybridomas to immortalize MBCs was first described over 40 years ago (39). Hybridomas are made by fusing a myeloma cell with a B-cell from an immunized or naturally infected individual, the resulting hybrid cell secretes mAbs specific to their antigen. Technical advances have made it feasible to generate hybridomas from human peripheral blood MBCs (40). These advances include expanding B-cells prior to fusion, finding new human myeloma cells to fuse with, and improved fusion techniques including electrical cytofusion (41). Using optimized techniques Yu et al. (41), fusion efficiency improved from 0.001 (40) to 0.43% (41) which enabled them to isolate neutralizing mAbs against RSV and influenza from human peripheral MBCs. Hybridoma technology is a well-established and indispensable platform for generating high-quality mAbs and has been used to produce mAbs against a wide range of viral antigens including DENV.

### Strengths and Limitations

Major advantages of this approach include pairing of BCR heavy and light chains (42), native constant region of the mAb expressed allowing Fc-mediated effector functions, such as enhancement to be accessed (40). Finally, the hybridoma products are stable in culture and can be frozen for future use.

One major limitation of this approach is extremely low fusion efficiency. Consequently, traditional hybridoma strategies are not as well suited for identifying rare antigen-specific MBCs that circulate in low numbers in the periphery of immune donors, as overall only a small amount of the total B cell repertoire is captured.

The second major limitation has been the challenge of making human, rather than mouse, derived hybridomas. Work by Wahala et al. (43) found that humans and mice recognize distinct and different epitopes on the DENV virion following immunization in mice or natural infection in humans. Nearly all neutralizing antibodies found in humans after natural infection recognize complex quaternary epitopes on the surface of whole virions (44, 45), in contrast to the DENV neutralizing antibody response in mice, where the majority of neutralizing antibodies recognize a single domain region, domain III, on the envelope glycoprotein (43).

### B-Cell Immortalization

MBC immortalization can be achieved through transforming peripheral MBCs using Epstein Barr Virus (EBV), or through expression of BCL-6, and BCL-XL. This results in stable cell lines that express BCR on the surface and secrete antibodies, making them a useful tool in the generation of human mAbs and has become a leading approach in characterizing DENV-specific MBCs in humans.

EBV transformation for B-cell cultures was developed in the 1970's Steinitz et al. (46) when normal human B-cells were infected with EBV, a lymphotropic herpesvirus, transforming MBCs into stable antibody secreting cell lines. Supernatants can be screened for specificity to antigen of choice and serial dilution down to a single cell enables this method to be applied to mAb production. Many groups have utilized and continue to utilize EBV immortalization to isolate human mAbs against a wide variety of human pathogens, including HIV (47), SARS coronavirus (48), Influenza (49), RSV (50), and DENV (14, 15, 51).

Another technique employed to immortalize MBCs is through forced expression of BCL-6 (required for GC formation) and BCL-XL (anti-apoptotic Bcl-2 protein family). Both are expressed in GC B-cells, and by introducing these genes into peripheral blood MBCs and culturing with CD40L and IL-21, they become highly proliferating with surface and secreted Ig (52). BCL-6 + BCL-XL transduced cells express *AICDA*, encoding the enzyme activation-induced cytidine deaminase (AID), at the same levels as isolated tonsil derived GC B-cells, but not normally expressed in peripheral MBCs or plasma cells. AID mediates somatic hypermutation (SHM) and class-switch recombination (CSR) and therefore increases diversity of the BCR. AID is functional in these cells and low levels of SHM is observed in the Ig genes of expanded B-cells. These cells can be maintained for prolonged periods of time in culture to allow for mAb production (53). Using this approach, researchers have identified neutralizing mAbs in humans that recognize RSV (52), Hepatitis C virus (54), influenza (55), and DENV (56).

### Strengths and Limitations

Immortalized B-cells have a plasmablast-like phenotype, with secretory and membrane-bound Ig, which makes them a powerful tool for discovery and characterization of mAbs. Probes that bind to BCRs of interest enable the isolation of antigen-specific B-cells from a polyclonal population. Immortalized cells are stable and can be frozen for future use. The presence of AID and the potential for SHM can be utilized to generate clones that have higher or lower affinities than the parental clone, allowing for a method of affinity maturation in culture (53).

Transformation efficiency for BCL-6+BCL-XL is 60–80% in humans (53), and EBV transformation have improved from 10 to >30% with the addition of TLR agonists, typically CpG or R848 (50, 57). This approach requires significant numbers of cells to yield few cells of interest. Because cells proliferate with this approach, frequency of particular antigen-specific MBCs cannot be enumerated.

Using EBV-transformed human B-cells to generate human hybridomas can increase efficiency by as much as 25-fold compared to that of using untransformed PBMCs. Therefore, Investigators often utilize a combination approach of EBV immortalization followed by fusion to isolate human DENV-specific mAbs from naturally infected or vaccinated donors (28, 55).

### Antigen—Specific Flow Cytometry

Flow cytometry-based approaches have been used to enumerate antigen specific MBCs against model antigens in mice (58) and humans (33). However, viral antigen-specific flow cytometry has been utilized more recently, Weitkamp et al. (59) identified human rotavirus specific B-cells, Scheid et al. (60) characterized low-frequency HIV specific MBCs in humans and Woda et al. (61) characterized DENV-specific MBCs in human immune donors. Recognizing the complex and quaternary nature of DENV neutralizing epitopes (45) Authors Woda and Mathew (26) and Appanna et al. (28) used fluorescently labeled whole DENV virus (62) as a probe to detect DENV-specific MBCs in immune donors while Cox et al. (25) used biotinylated DENV envelope protein as a probe along with dual labeled streptavidin antibodies to identify DENV envelope-specific MBCs. This method enabled researchers to isolate 8 DENV-neutralizing mAbs from a single donor (Table 1).

### Strengths and Limitations

Antigen choice is important, DENV neutralizing epitopes are comprised of complex conformational structures and not recapitulated by simple linear peptides or recombinant proteins (45). However, whole viruses are inherently sticky and adheres to host cells. To tackle this non-specific binding (26, 61) utilized fluorescently labeled Vero cell supernatant as well as dual labeled probes to decrease background (63). A major strength of this approach is the possibility of tracking multiple serotypes of DENV- specific MBCs prior to and post infection or vaccination (61) as well as the potential for single cell sorting antigen-specific MBCs for downstream assays such as immortalization, sequencing, or cloning (25).

## Future Directions

An important early advancement in the field of human mAb generation was the advent of single-cell RT-PCR approaches (64) that allow for sequencing, cloning, and characterization of each BCR from individually sorted MBCs. This approach remains useful when the population of interest represents a large proportion of total cells in population (plasmablasts during acute infection), or when a valid probe or screening approach exists to identify MBCs of interest prior to sequencing. In addition to generating mAbs, sequencing of the BCR provides information about B-cell clonal evolution during infection. While groundbreaking, this single-cell approach is time and resource intensive as it requires heavy and light chains to undergo PCR, sequencing, and cloning independently and remain correctly paired for transfection into expression plasmids.

This single-cell approach provides a glimpse into the overall antibody repertoire, which has a potential diversity of more than 1 × 10^13^ in humans, but high throughput methods that capture the entire antigen-specific MBC repertoire recently developed with other pathogens would be expected to advance the DENV field as well. High-throughput droplet microfluidic approaches (65) allow for individual partitioning of single B-cells, that are individually barcoded and allow for paired sequencing of Ig heavy and light chains from a single B cell captured within a droplet. From this, a complete Ig library can be generated, as well this approach allows for simultaneous sequencing of barcoded Ig genes with the possibility of co-expressed functional genes to fully understand the pathogen specific MBC repertoire. MAbs that are generated from these antibody gene sequences allow for a functional analysis of the repertoire.

Another high throughput approach (66, 67) recently used to isolate mAbs from humans involves using microfluidics to partition individual cells then physically link heavy and light mRNAs and perform overlap extension PCR to generate a continuous heavy-light chain amplicon for cloning into a yeast display system for Fab or IgG which allows screening for antigen specificity and affinity by FACS. Through this approach researchers were able to isolate broadly neutralizing antibodies against HIV, Ebola, and influenza.

The ability to fully interrogate the MBC response established after natural infection to viral antigens will allow researchers to durably and comprehensively interrogate vaccine responses to further understand the differences between natural and vaccine derived immunity.

## Author Contributions

All authors listed have made a substantial, direct and intellectual contribution to the work, and approved it for publication.

### Conflict of Interest Statement

The authors declare that the research was conducted in the absence of any commercial or financial relationships that could be construed as a potential conflict of interest.

## References

[1] Slifka MK Antia R Whitmire JK Ahmed R. Humoral immunity due to long-lived plasma cells. Immunity. (1998) 8:363–72. 10.1016/S1074-7613(00)80541-5 9529153

[2] Amanna IJ Slifka MK. Mechanisms that determine plasma cell lifespan and the duration of humoral immunity. Immunol Rev. (2010) 236:125–38. 10.1111/j.1600-065X.2010.00912.x 20636813 PMC7165522

[3] Yoshida T Mei H Dorner T Hiepe F Radbruch A Fillatreau S. Memory B and memory plasma cells. Immunol Rev. (2010) 237:117–39. 10.1111/j.1600-065X.2010.00938.x 20727033

[4] Bernasconi NL Traggiai E Lanzavecchia A. Maintenance of serological memory by polyclonal activation of human memory B cells. Science. (2002) 298:2199–202. 10.1126/science.1076071 12481138

[5] Inoue T Moran I Shinnakasu R Phan TG Kurosaki T. Generation of memory B cells and their reactivation. Immunol Rev. (2018) 283:138–49. 10.1111/imr.12640 29664566

[6] Amanna IJ Carlson NE Slifka MK. Duration of humoral immunity to common viral and vaccine antigens. N Engl J Med. (2007) 357:1903–15. 10.1056/NEJMoa066092 17989383

[7] Purtha WE Tedder TF Johnson S Bhattacharya D Diamond MS Memory B cells, but not long-lived plasma cells, possess antigen specificities for viral escape mutants. J Exp Med. (2011) 208:2599–606. 10.1084/jem.20110740 22162833 PMC3244041

[8] Seifert M Przekopowitz M Taudien S Lollies A Ronge V Drees B. Functional capacities of human IgM memory B cells in early inflammatory responses and secondary germinal center reactions. Proc Natl Acad Sci USA. (2015) 112:E546–55. 10.1073/pnas.1416276112 25624468 PMC4330750

[9] Wienands J Engels N. The Memory Function of the B Cell Antigen Receptor. Curr Top Microbiol Immunol. (2016) 393:107–21. 10.1007/82_2015_480 26362935

[10] Shinnakasu R Kurosaki T. Regulation of memory B and plasma cell differentiation. Curr Opin Immunol. (2017) 45:126–31. 10.1016/j.coi.2017.03.003 28359033

[11] Moir S Fauci AS. B-cell responses to HIV infection. Immunol Rev. (2017) 275:33–48. 10.1111/imr.12502 28133792 PMC5300048

[12] Cortjens B Yasuda E Yu X Wagner K Claassen YB Bakker AQ. Broadly reactive anti-respiratory syncytial virus G antibodies from exposed individuals effectively inhibit infection of primary airway epithelial cells. J Virol. (2017) 91:e02357–16. 10.1128/JVI.02357-16 28275185 PMC5411575

[13] Wrammert J Smith K Miller J Langley WA Kokko K Larsen C. Rapid cloning of high-affinity human monoclonal antibodies against influenza virus. Nature. (2008) 453:667–71. 10.1038/nature06890 18449194 PMC2515609

[14] Beltramello M Williams KL Simmons CP Macagno A Simonelli L Quyen NT. The human immune response to Dengue virus is dominated by highly cross-reactive antibodies endowed with neutralizing and enhancing activity. Cell Host Microbe. (2010) 8:271–83. 10.1016/j.chom.2010.08.007 20833378 PMC3884547

[15] Schieffelin JS Costin JM Nicholson CO Orgeron NM Fontaine KA Isern S. Neutralizing and non-neutralizing monoclonal antibodies against dengue virus E protein derived from a naturally infected patient. Virol J. (2010) 7:28. 10.1186/1743-422X-7-28 20132551 PMC2829534

[16] Bhatt S Gething PW Brady OJ Messina JP Farlow AW Moyes CL. The global distribution and burden of dengue. Nature. (2013) 496:504–7. 10.1038/nature12060 23563266 PMC3651993

[17] Vasilakis N Weaver SC. The history and evolution of human dengue emergence. Adv Virus Res. (2008) 72:1–76. 10.1016/S0065-3527(08)00401-6 19081488

[18] Katzelnick LC Coloma J Harris E. Dengue: knowledge gaps, unmet needs, and research priorities. Lancet Infect Dis. (2017) 17:e88–100. 10.1016/S1473-3099(16)30473-X 28185868 PMC5967882

[19] Andrade P Coloma J Harris E. ELISPOT-based “multi-color fluorospot” to study type-specific and cross-reactive responses in memory b cells after dengue and zika virus infections. Methods Mol Biol. (2018) 1808:151–63. 10.1007/978-1-4939-8567-8_13 29956181

[20] Halstead SB O'Rourke EJ. Antibody-enhanced dengue virus infection in primate leukocytes. Nature. (1977) 265:739–41. 10.1038/265739a0 404559

[21] Gallichotte EN Baric RS de Silva AM. The molecular specificity of the human antibody response to dengue virus infections. Adv Exp Med Biol. (2018) 1062:63–76. 10.1007/978-981-10-8727-1_5 29845525

[22] Tsai WY Chen HL Tsai JJ Dejnirattisai W Jumnainsong A Mongkolsapaya J. Potent neutralizing human monoclonal antibodies preferentially target mature dengue virus particles: implication for novel strategy for dengue vaccine. J Virol. (2018) 92:e00556–18. 10.1128/JVI.00556-18 30185598 PMC6232466

[23] Raut R Corbett KS Tennekoon RN Premawansa S Wijewickrama A Premawansa G. Dengue type 1 viruses circulating in humans are highly infectious and poorly neutralized by human antibodies. Proc Natl Acad Sci USA. (2019) 116:227–32. 10.1073/pnas.1812055115 30518559 PMC6320508

[24] Wahala WM Silva AM. The human antibody response to dengue virus infection. Viruses. (2011) 3:2374–95. 10.3390/v3122374 22355444 PMC3280510

[25] Cox KS Tang A Chen Z Horton MS Yan H Wang X-M. Rapid isolation of dengue-neutralizing antibodies from single cell-sorted human antigen-specific memory B-cell cultures. MAbs. (2015) 8:129–40. 10.1080/19420862.2015.1109757 26491897 PMC4966506

[26] Woda M Mathew A. Fluorescently labeled dengue viruses as probes to identify antigen-specific memory B cells by multiparametric flow cytometry. J Immunol Methods. (2015) 416:167–77. 10.1016/j.jim.2014.12.001 25497702 PMC4440233

[27] Smith SA Zhou Y Olivarez NP Broadwater AH de Silva AM Crowe JE Jr. Persistence of circulating memory B cell clones with potential for dengue virus disease enhancement for decades following infection. J Virol. (2012) 86:2665–75. 10.1128/JVI.06335-11 22171265 PMC3302281

[28] Appanna R Kg S Xu MH Toh YX Velumani S Carbajo D. Plasmablasts during acute dengue infection represent a small subset of a broader virus-specific memory B cell pool. EBioMedicine. (2016) 12:178–88. 10.1016/j.ebiom.2016.09.003 27628668 PMC5078588

[29] Smith SA de Alwis R Kose N Durbin AP Whitehead SS de Silva AM. Human monoclonal antibodies derived from memory B cells following live attenuated dengue virus vaccination or natural infection exhibit similar characteristics. J Infect Dis. (2013) 207:1898–908. 10.1093/infdis/jit119 23526830 PMC3654755

[30] Smith SA de Alwis AR Kose N Jadi RS de Silva AM Crowe JE Jr. (2014). Isolation of dengue virus-specific memory B cells with live virus antigen from human subjects following natural infection reveals the presence of diverse novel functional groups of antibody clones. J Virol. (2014) 88:12233–41. 10.1128/JVI.00247-14 25100837 PMC4248927

[31] Nivarthi UK Kose N Sapparapu G Widman D Gallichotte E Pfaff JM. Mapping the Human memory B cell and serum neutralizing antibody responses to dengue virus serotype 4 infection and vaccination. J Virol. (2017) 91: e02041–16. 10.1128/JVI.02041-16 28031369 PMC5309932

[32] Slifka MK Ahmed R. Limiting dilution analysis of virus-specific memory B cells by an ELISPOT assay. J Immunol Methods. (1996) 199:37–46. 10.1016/S0022-1759(96)00146-9 8960096

[33] Amanna IJ Slifka MK. Quantitation of rare memory B cell populations by two independent and complementary approaches. J Immunol Methods. (2006) 317:175–85. 10.1016/j.jim.2006.09.005 17055526 PMC1850941

[34] Pinna D Corti D Jarrossay D Sallusto F Lanzavecchia A. Clonal dissection of the human memory B-cell repertoire following infection and vaccination. Eur J Immunol. (2009) 39:1260–70. 10.1002/eji.200839129 19404981 PMC3864550

[35] Narvaez CF Feng N Vasquez C Sen A Angel J Greenberg HB. Human rotavirus-specific IgM Memory B cells have differential cloning efficiencies and switch capacities and play a role in antiviral immunity *in vivo*. J Virol. (2012) 86:10829–40. 10.1128/JVI.01466-12 22855480 PMC3457288

[36] Czerkinsky CC Nilsson LA Nygren H Ouchterlony O Tarkowski A. A solid-phase enzyme-linked immunospot (ELISPOT) assay for enumeration of specific antibody-secreting cells. J Immunol Methods. (1983) 65:109–21. 10.1016/0022-1759(83)90308-3 6361139

[37] Sasaki S Jaimes MC Holmes TH Dekker CL Mahmood K Kemble GW Comparison of the influenza virus-specific effector and memory B-cell responses to immunization of children and adults with live attenuated or inactivated influenza virus vaccines. J Virol. (2007) 81:215–28. 10.1128/JVI.01957-06 17050593 PMC1797237

[38] Adam A Woda M Kounlavouth S Rothman AL Jarman RG Cox JH. Multiplexed fluorospot for the analysis of dengue virus- and zika virus-specific and cross-reactive memory B cells. J Immunol. (2018) 201:3804–14. 10.4049/jimmunol.1800892 30413671 PMC6289764

[39] KÖHler G Milstein C. Continuous cultures of fused cells secreting antibody of predefined specificity. Nature. (1975) 256:495–7. 10.1038/256495a0 1172191

[40] Smith SA Crowe JE Jr. Use of human hybridoma technology to isolate human monoclonal antibodies. Microbiol Spectr. (2015) 3:Aid-0027-2014. 10.1128/microbiolspec.AID-0027-2014 26104564 PMC8162739

[41] Yu X McGraw PA House FS Crowe JE Jr. An optimized electrofusion-based protocol for generating virus-specific human monoclonal antibodies. J Immunol Methods. (2008) 336:142–51. 10.1016/j.jim.2008.04.008 18514220 PMC2519117

[42] Liu JK. The history of monoclonal antibody development - Progress, remaining challenges and future innovations. Ann Med Surg. (2014) 3:113–6. 10.1016/j.amsu.2014.09.001 25568796 PMC4284445

[43] Wahala WM Kraus AA Haymore LB Accavitti-Loper MA de Silva AM. Dengue virus neutralization by human immune sera: role of envelope protein domain III-reactive antibody. Virology. (2009) 392:103–13. 10.1016/j.virol.2009.06.037 19631955 PMC2746956

[44] de Alwis R Smith SA Olivarez NP Messer WB Huynh JP Wahala WM. Identification of human neutralizing antibodies that bind to complex epitopes on dengue virions. Proc Natl Acad Sci USA. (2012) 109:7439–44. 10.1073/pnas.1200566109 22499787 PMC3358852

[45] Dejnirattisai W Wongwiwat W Supasa S Zhang X Dai X Rouvinski A A new class of highly potent, broadly neutralizing antibodies isolated from viremic patients infected with dengue virus. Nat Immunol. (2015) 16:170–7. 10.1038/ni.3058 25501631 PMC4445969

[46] Steinitz M Klein G Koskimies S Makel O. EB virus-induced B lymphocyte cell lines producing specific antibody. Nature. (1977) 269:420–2. 10.1038/269420a0 198669

[47] Robinson JE Holton D Pacheco-Morell S Liu J McMurdo H. Identification of conserved and variant epitopes of human immunodeficiency virus type 1 (HIV-1) gp120 by human monoclonal antibodies produced by EBV-transformed cell lines. AIDS Res Hum Retroviruses. (1990) 6:567–79. 10.1089/aid.1990.6.567 1694449

[48] Traggiai E Becker S Subbarao K Kolesnikova L Uematsu Y Gismondo MR. An efficient method to make human monoclonal antibodies from memory B cells: potent neutralization of SARS coronavirus. Nat Med. (2004) 10:871–5. 10.1038/nm1080 15247913 PMC7095806

[49] Simmons CP Bernasconi NL Suguitan AL Mills K Ward JM Chau NV. Prophylactic and therapeutic efficacy of human monoclonal antibodies against H5N1 influenza. PLoS Med. (2007) 4:e178. 10.1371/journal.pmed.0040178 17535101 PMC1880850

[50] Corti D Lanzavecchia A. Efficient methods to isolate human monoclonal antibodies from memory B cells and plasma cells. Microbiol Spectr. (2015) 2:129–139. 10.1128/microbiolspec.AID-0018-2014 26104354

[51] Friberg H Jaiswal S West K O'Ketch M Rothman AL Mathew A. Analysis of human monoclonal antibodies generated by dengue virus-specific memory B cells. Viral Immunol. (2012) 25:348–59. 10.1089/vim.2012.0010 22934599 PMC3466914

[52] Kwakkenbos MJ Diehl SA Yasuda E Bakker AQ van Geelen CM Lukens MV. Generation of stable monoclonal antibody-producing B cell receptor-positive human memory B cells by genetic programming. Nat Med. (2010) 16:123–8. 10.1038/nm.2071 20023635 PMC2861345

[53] Kwakkenbos MJ van Helden PM Beaumont T Spits H. Stable long-term cultures of self-renewing B cells and their applications. Immunol Rev. (2016) 270:65–77. 10.1111/imr.12395 26864105 PMC4755196

[54] Merat SJ Molenkamp R Wagner K Koekkoek SM van de Berg D Yasuda E. Hepatitis C virus broadly neutralizing monoclonal antibodies isolated 25 years after spontaneous clearance. PLoS ONE. (2016) 11:e0165047. 10.1371/journal.pone.0165047 27776169 PMC5077102

[55] Ekiert DC Friesen RH Bhabha G Kwaks T Jongeneelen M Yu W. A highly conserved neutralizing epitope on group 2 influenza A viruses. Science. (2011) 333:843–50. 10.1126/science.1204839 21737702 PMC3210727

[56] Nivarthi UK Tu HA Delacruz MJ Swanstrom J Patel B Durbin AP. Longitudinal analysis of acute and convalescent B cell responses in a human primary dengue serotype 2 infection model. EBioMedicine. (2019) 41:465–78. 10.1016/j.ebiom.2019.02.060 30857944 PMC6444124

[57] Lanzavecchia A. Dissecting human antibody responses: useful, basic and surprising findings. EMBO Mol Med. (2018) 10:e8879. 10.15252/emmm.201808879 29363490 PMC5840544

[58] Kodituwakku AP Jessup C Zola H Roberton DM. Isolation of antigen-specific B cells. Immunol Cell Biol. (2003) 81:163–70. 10.1046/j.1440-1711.2003.01152.x 12752679

[59] Weitkamp JH Kallewaard N Kusuhara K Feigelstock D Feng N Greenberg HB. Generation of recombinant human monoclonal antibodies to rotavirus from single antigen-specific B cells selected with fluorescent virus-like particles. J Immunol Methods. (2003) 275:223–37. 10.1016/S0022-1759(03)00013-9 12667686

[60] Scheid JF Mouquet H Feldhahn N Walker BD Pereyra F Cutrell E. A method for identification of HIV gp140 binding memory B cells in human blood. J Immunol Methods. (2009) 343:65–7. 10.1016/j.jim.2008.11.012 19100741 PMC2754789

[61] Woda M Friberg H Currier JR Srikiatkhachorn A Macareo LR Green S. Dynamics of Dengue Virus (DENV)-specific B cells in the response to DENV serotype 1 infections, using flow cytometry with labeled virions. J Infect Dis. (2016) 214:1001–9. 10.1093/infdis/jiw308 27443614 PMC5021233

[62] Zhang SL Tan HC Hanson BJ Ooi EE. A simple method for Alexa Fluor dye labelling of dengue virus. J Virol Methods. (2010) 167:172–7. 10.1016/j.jviromet.2010.04.001 20399231

[63] Moody MA Haynes BF. Antigen-specific B cell detection reagents: use and quality control. Cytometry A. (2008) 73:1086–92. 10.1002/cyto.a.20599 18613115 PMC3105373

[64] Tiller T Meffre E Yurasov S Tsuiji M Nussenzweig MC Wardemann H. Efficient generation of monoclonal antibodies from single human B cells by single cell RT-PCR and expression vector cloning. J Immunol Methods. (2008) 329:112–24. 10.1016/j.jim.2007.09.017 17996249 PMC2243222

[65] Wen N Zhao Z Fan B Chen D Men D Wang J. Development of droplet microfluidics enabling high-throughput single-cell analysis. Molecules. (2016) 21:881. 10.3390/molecules21070881 27399651 PMC6272933

[66] Adler AS Mizrahi RA Spindler MJ Adams MS Asensio MA Edgar RC. Rare, high-affinity anti-pathogen antibodies from human repertoires, discovered using microfluidics and molecular genomics. MAbs. (2017) 9:1282–96. 10.1080/19420862.2017.1371383 28846502 PMC5680809

[67] Wang B DeKosky BJ Timm MR Lee J Normandin E Misasi J. Functional interrogation and mining of natively paired human VH:VL antibody repertoires. Nat Biotechnol. (2018) 36:152–5. 10.1038/nbt.4052 29309060 PMC5801115

